# Open abdomen management and outcomes: two case reports from western Kenya and a review of literature from Africa

**DOI:** 10.11604/pamj.2019.32.33.17859

**Published:** 2019-01-17

**Authors:** Clifford Mwita, Ruth Negesa, Marissa Boeck, Andrew Wandera

**Affiliations:** 1 Department of Surgery and Anesthesiology, Moi University School of Medicine, Eldoret, Kenya; 2 Afya Research Africa, Nairobi, Kenya; 3 Department of Surgery, New York-Presbyterian Hospital/Columbia, New York, United State of America

**Keywords:** Open abdomen, laparostomy, Sub-Saharan Africa

## Abstract

The open abdomen (OA) is clinically indicated for attenuating the effects of select intra-abdominal insults that may lead to high intra-abdominal pressure with fascial closure. Despite the high incidence of conditions warranting OA in Africa, there are few reports on its use and outcomes. A retrospective chart review was performed for two patients managed with an OA at the Moi Teaching and Referral Hospital. For comparison, a literature review on related studies from Africa was performed. One patient had an anastomotic leak, while the other had a perforated gastric ulcer. A Bogotá bag was used for temporary abdominal content containment. There was no mortality in our series and fascial closure was achieved in one patient. Upon review of studies from Africa, overall mortality stood at 44%, while 25% of surviving patients underwent fascial closure. The use of OA in Africa is associated with high mortality and low rates of fascial closure. Our limited experience shows this technique is a viable treatment option in an attempt to bridge a patient to abdominal closure during critical illness.

## Introduction

The open abdomen (OA) or laparostomy is defined as the intentional separation of the cutaneous and musculofascial layers of the abdominal wall [1]. Since its inception, it has steadily gained acceptance among surgeons as a means of attenuating the effects of select life-threatening abdominal insults that lead to intra-abdominal hypertension (IAH) and the development of abdominal compartment syndrome (ACS). Both globally and in Africa, the commonest insults warranting an OA are abdominal trauma and sepsis [2]. Between 17.4% and 25% of patients with these conditions in Africa have IAH on admission, and mortality varies from 2.4% to 24.4% [3, 4]. Nonetheless, there is ample evidence that control of IAH through the use of the OA mitigates the potentially lethal effects of ACS [5]. The main goal after the creation of an OA is fascial closure as soon as the underlying insult has resolved [1]. However, in the interim, there is a need for temporary abdominal closure (TAC), which is fraught with multiple local and systemic complications. TAC techniques vary, and each carries its own set of advantages and disadvantages. As such, managing OA is resource intensive and a multidisciplinary approach is often warranted. The intensive care unit (ICU) may be needed for ventilatory support, correction of coagulopathy, fluid, electrolyte and acid/base disorders, as well as prevention of hypothermia [5]. In addition, there is a need for appropriate antibiotic therapy, pain control, and sedation, with many patients requiring paralysis throughout their course to avoid evisceration. Patients are often hypercatabolic and require supplemental nutritional support and intensive nursing care. Although the use of the OA in contemporary surgical practice is widely accepted, there is a paucity of information from Africa regarding its use and subsequent outcomes. A single center study done nearly two decades ago reported abdominal trauma and abdominal sepsis as the commonest reasons for an OA, with a low rate of fascial closure and mortality as high as 44% [2]. More recent data corroborates the high prevalence of abdominal trauma and sepsis in Africa [4, 6, 7]. However, the use of the OA in the care and subsequent outcome of these conditions remains obscure, and more studies are warranted. We report the experience of using the OA technique in a tertiary care institution in western Kenya. Additionally, we performed a literature search for similar studies from Africa with the aim of documenting OA outcomes, and comparing our experience with those from comparable settings.

## Patient and observation

**Figure 1:**
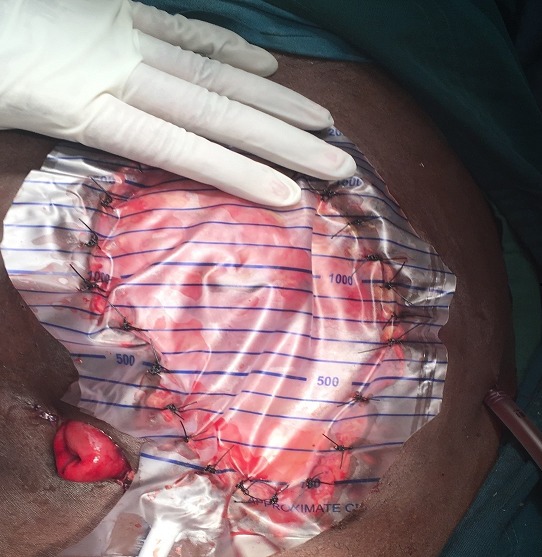
Bogota bag fashioned from urine bag and used for temporary abdominal content containment

**Figure 2:**
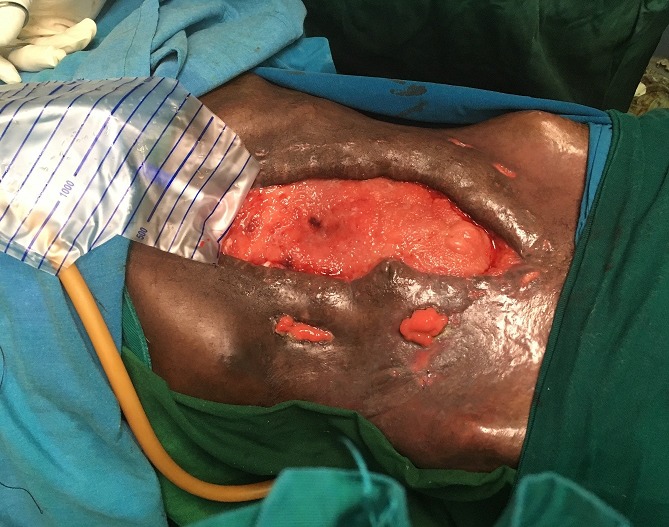
Abdominal wound with formation of granulation tissue

**Case 1**: A 34-year-old man presented to the Moi Teaching and Referral Hospital (MTRH) in Eldoret, Kenya with a history of leaking fecal matter from a midline abdominal incision used for two laparotomies at an outside hospital. The remainder of his history was unremarkable except for the use of alcohol and tobacco. On examination, he appeared ill, pale, tachycardic and tachypneic, but was normotensive. His abdomen was distended with a midline laparotomy incision leaking copious amounts of fecal matter. His hemoglobin and hematocrit levels were low. There was a slight leukocytosis with neutrophilia and normal platelet counts. Urea, creatinine and electrolytes were within normal limits. After resuscitation, he was taken to theatre for a laparotomy, where the peritoneal cavity was found to be completely soiled with fecal matter that was leaking from two anastomotic sites from his previous surgeries. The leaking segments of gut were resected, an abdominal washout was done, and an ileostomy was fashioned. However, due to extensive bowel edema, it was not possible to achieve fascial closure and a Bogotá bag, fashioned out of a recommissioned catheter drainage bag or intravenous fluid bag, was used for TAC (Figure 1). The absence of space in the ICU meant his care was to be continued in the general surgical ward. During his post-operative course, the patient underwent five re-look laparotomies with abdominal washouts for abdominal abscesses, and one instance for a colonic perforation at the hepatic flexure requiring a right colectomy. Each time, fascial closure was not feasible and a Bogotá bag was used for TAC. A central venous catheter was placed for fluid and antibiotic therapy. A Foley catheter was used to monitor urine output, and a stoma bag was used for routine stoma care. Enteral feeds were started early and maintained throughout his hospital stay, although he became cachectic, at one point weighing only 37 kilograms. His abdominal wound was dressed daily and allowed to granulate (Figure 2). Despite the challenge of caring for the OA in the setting of an adjacent ileostomy, the patient did not develop an entero-atmospheric fistula. He spent a total of 121 days in hospital and was discharged after adequate weight gain, and contracture of his abdominal wound.

**Case 2**: A 21-year-old man with a prior history of dyspepsia presented to MTRH with a 3-day history of acute onset upper abdominal pain, accompanied by vomiting and abdominal distension. The remainder of his history was unremarkable. On examination, he was tachycardic, tachypneic, and hypotensive. His abdomen was distended, tender with guarding, and silent on auscultation. Although his blood counts were unremarkable, he had azotemia, hyponatremia, and hyperkalemia. An abdominal x-ray showed air under the right hemidiaphragm. After fluid resuscitation and correction of his electrolyte abnormalities, he was taken to theatre with a diagnosis of perforated viscus. Upon entering the peritoneal cavity, more than 2 liters of succus entericus was found emanating from a gastric wall perforation. A biopsy was taken, and an omental (Graham) patch was used to repair the defect. However, fascial closure could not be achieved owing to extensive bowel edema, and a Bogotá bag was used for TAC. The patient remained stable and a second-look laparotomy was performed 48 hours later. The gastric repair was still intact, bowel edema had subsided, and fascial closure was successful. He was discharged on a proton pump inhibitor one week later after spending a total of 10 days in the hospital. Histology was benign and the patient remained symptom free at the follow-up clinic.

## Discussion

A review of the literature was performed via PubMed and African Journals Online (AJOL) for studies from Africa that described both the management and outcome of patients with OA for any underlying reason. There were no date restrictions and databases were searched from inception to March 2018. The search terms used for each database are shown in Table 1. Out of a total of 805 identified studies, 796 were excluded because they did not meet the aforementioned inclusion criteria. The remaining 9 studies [2, 6, 8-14] were considered for inclusion. Table 2 summarizes data on patient characteristics, method of TAC, and the outcomes of interest (mortality and fascial closure). Similar data from the present series were also included for comparison. Overall, a total of N=268 adults and N=15 neonates were included. The mortality rate among adults was 44%, versus 20% among neonates. A total of N=67 adult patients had fascial closure translating to a rate of 25%. Twelve (80%) of neonates had fascial closure. Other complications included inability of the TAC method to contain abdominal contents leading to evisceration i.e. TAC failure (N=2 adult patients) and intestinal fistula formation (N=3 neonates).

**Table 1:** Search strategy

Database	Search terms	Results
PubMed	(((((Africa) OR sub Saharan Africa) OR Africa [MeSH Terms]) OR sub Saharan Africa [MeSH Terms])) AND (((((abbreviated laparotomy) OR damage control laparotomy) OR damage control surgery)) OR (((((((((((((open abdomen) OR laparostomy) OR wound dehiscence) OR open peritoneal cavity) OR celiotomy) OR open management abdomen) OR open abdominal wound) OR abdominal wall defect)) OR temporary abdominal content containment) OR temporary abdominal closure) OR abdominal compartment syndrome) OR intra-abdominal hypertension))	797
African Journals Online (AJOL)	Open abdomen	4
	laparostomy	4

**Table 2:** Characteristics of reviewed studies

Author/Date	Country	Patient characteristics	Open abdomen technique and treatment	Outcomes
Ghimenton 2000 [2]	South Africa	157 adult patients with intra-abdominal sepsis or abdominal trauma	Polyglactin mesh or Bogotá bag	69 deaths (44% mortality) 2 (1.3%) TAC^d^ failure 28 (18%) fascial closure
Gisore 2013 [6]	Kenya	5 adult patients with wound dehiscence or intra-abdominal sepsis	NPWT^e^ using gauze	0 deaths (0% mortality) 5 (100%) fascial closure
Chamisa 2008 [8]	South Africa	30-year-old male with penetrating neck injury, shock and secondary abdominal compartment syndrome due to over-resuscitation	Decompression laparotomy with Bogotá bag and ICU care	Death from multiple organ failure syndrome
Hattori 2017 [9]	South Africa	15 neonates with various abdominal surgical conditions	VAC after surgery followed by ICU care	3 deaths (20% mortality) 1 (6.7%) fistula 12 (80%) fascial closure
Krige 2016 [10]	South Africa	6 adult patients with severe irreparable pancreatoduodenal injuries	Damage control laparotomy with open abdomen using modified VAC technique and ICU care	2 deaths (33% mortality) No report of fascial closure
Laing 2013 [11]	South Africa	8-year-old male with severe duodenal and biliary injury due to crush injury to the abdomen	Damage control laparotomy, ICU care and TPN Method of temporary closure not mentioned	Survived Fascial closure achieved
Mari 2013 [12]	Uganda	10 adult patients with diffuse peritonitis	Skin closure for temporary closure, intravenous fluids and antibiotics as well as daily peritoneal lavage via abdominal drain tubes No ICU care	3 deaths (30% mortality) 7 (70%) fascial closure
Navasaria 2003 [13]	South Africa	55 adult trauma patients	Modified sandwich-vacuum pack technique, ICU care	25 deaths (45% mortality) 3 (5.4%) fistulas 16 (29%) fascial closure
Schein 1991 [14]	South Africa	31 adult patients with severe intra-abdominal infections	VAC^a^, ICU^b^ care and TPN^c^ until return of bowel function	18 deaths (58% mortality) 5 (16%) fascial closure
Present report	Kenya	2 adult patients with intra-abdominal sepsis	Bogota bag for TAC, intravenous fluids, antibiotics and enteral nutrition as early as feasible	0 (0% mortality) 1 (50%) Fascial closure

a Vacuum assisted closure

b Intensive care unit

c Total parenteral nutrition

d Temporary abdominal closure

e Negative pressure wound therapy

In contemporary surgical practice, the use of the OA to diminish the deleterious effects of IAH/ACS is widely accepted. However, in as much as the OA may be lifesaving, historically it is a resource intensive undertaking with high mortality, and morbidities that may affect the quality of life of involved patients. Despite the high prevalence of IAH/ACS among general surgery patients in Africa, the indications, use, and outcomes of the OA in the region remain inadequately documented. From this report, the commonest reasons for utilizing OA management in the African region are abdominal trauma and sepsis. Both of these conditions are fairly prevalent and often warrant treatment in a critical care unit [3, 15]. Nonetheless, the majority of published reports are from one upper-middle income African country, and as such, experiences from other parts of Africa remain unknown. The present series from western Kenya presents two adult patients with severe intra-abdominal infection following gastro-intestinal perforation. In both cases, there was no measurement of pre-operative intra-abdominal pressure (IAP), even though it is recommended in patients suspected of intra-abdominal sepsis [5]. The recommended measurement methods are not feasible at our institution. It is therefore prudent to always keep in mind the possibility of IAH/ACS in all patients with suspected severe intra-abdominal infection. Despite the dangers of IAH/ACS, routine use of an OA for management of abdominal sepsis is not recommended, and other measures should be instituted before the OA is considered [5]. These include nasogastric/colonic decompression, avoiding positive fluid balance, sedation and analgesia, and damage control resuscitation [5]. Proper patient selection for OA management is imperative, which must include informed consent that covers prognosis and estimated cost of care. This is particularly important in resource limited areas where surgical care may lead to individual financial catastrophe.

Our patients were managed using the Bogotá bag. However, this technique is associated with frequent and time-consuming dressing changes, intensive nursing care, and prolonged treatment before definitive wound closure, all of which negatively impact quality of life. For these reasons, TAC techniques that employ negative wound pressure are recommended [5]. In addition, patients with OAs typically require ICU care, strict monitoring of fluid balance, and supplemental nutrition. In the present series, however, care was entirely provided in the general surgical ward due to a lack of ICU space (neither patient could afford ICU care, and there were no ICU beds available). Both were able to tolerate enteral feeds early in their clinical course, and therefore did not require total parenteral nutrition. Although we record no mortality, fascial closure could only be achieved in one patient. In a similar series involving a heterogenous group of five patients managed with OAs from another tertiary institution in Kenya, Gisore and colleagues reported the use of negative pressure wound therapy (NPWT) for TAC with no mortality and complete fascial closure in all patients [6]. All of the studies identified from the literature search were either from southern or eastern Africa. A variety of methods were used for TAC but the majority utilized negative pressure as recommended in the literature [16]. However, it is worth mentioning that the majority of the studies were from South Africa, which is classified by the World Bank as an upper middle-income country [13]. As such, direct comparison with other African countries may be misleading. Although this report reveals abdominal sepsis and trauma as the leading causes of OAs in Africa, in western countries, vascular disorders such as ruptured aortic aneurysms and mesenteric ischemia are other causes of IAH/ACS for which the OA is utilized [16].

The mortality attributable to OA in Africa is higher than in high-income countries. This is likely due to multiple variables, including the severity of the underlying condition warranting OA management, rather than solely the OA management itself. In a systematic review of delayed fascial closure for the OA, vanHensbroek and colleagues included studies from high-income countries, with an overall mortality of 26% [16]. Additionally, mortality was lower for studies that utilized negative pressure for TAC versus those that employed the Bogotá bag. The fascial closure rate for adults was 44.6% in this report. This means approximately half of the survivors did not have fascial closure and were left with ventral hernias. Although the OA may alleviate mortality, it is clear that quality of life can be severely affected. Other complications include development of intestinal fistulae and failure of TAC methods. Direct implications from this include prolonged hospital stays and higher medical costs. One study on the OA in the present report was done exclusively on newborns [9]. In this category, the observed mortality (20%) was lower than that seen in adults. However, it should be remembered that this is based on a single center series and therefore it may not reflect the true picture of OA management in this demographic in Africa. Although neonates had a higher rate of fascial closure, their complication rates were similar to those for adults. Further reports in neonate population would help determine whether the observed differences with adults are true. As a limitation, this report did not identify studies from other regions of Africa outside Sub-Saharan Africa (SSA). As such, the available picture is limited to eastern and southern Africa, and the results may lack external validity. It is likely that surgical units in other parts of the continent use the OA technique, but either do not document their experiences or their work is not easily identified due to poor indexing.

## Conclusion

The use of the OA in Africa is historically associated with high mortality and low rates of fascial closure, which likely affects quality of life among survivors. These outcomes undoubtedly are linked to the severity of the underlying abdominal insult, which is difficult to separate from the OA technique itself. With published data only reflecting a section of the region, more longitudinal studies from other settings across Africa are warranted. Only in this way can we truly understand and maximize the use of this treatment modality to improve the lives of patients suffering from abdominal catastrophes across varied income settings.

## Competing interests

The authors declare no competing interests.
